# P-1235. HPLC-UV method to establish rapid therapeutic drug monitoring of voriconazole in outpatients

**DOI:** 10.1093/ofid/ofae631.1417

**Published:** 2025-01-29

**Authors:** Yusuke Yagi, Kohei Jobu, Tomoaki Ishida, Narika Yanagisawa, Moemi Okazaki, Yu Arakawa, Yuka Yamagishi, Nobuaki Matsunaga, Yukihiro Hamada

**Affiliations:** Department of Infection Prevention and Control, Kochi Medical School Hospital, NANKOKU, Kochi, Japan; Department of Pharmacy, Kochi Medical School Hospital, NANKOKU, Kochi, Japan; Department of Pharmacy, Kochi Medical School Hospital, NANKOKU, Kochi, Japan; Department of Pharmacy, Kochi Medical School Hospital, NANKOKU, Kochi, Japan; Department of Pharmacy, Kochi Medical School Hospital, NANKOKU, Kochi, Japan; Department of Clinical Infectious Diseases, Kochi Medical School, NANKOKU, Kochi, Japan; Kochi University, Nankoku, Kochi, Japan; National Center for Global Health and Medicine, Shinjuku, Tokyo, Japan; Department of Pharmacy, Kochi Medical School Hospital, NANKOKU, Kochi, Japan

## Abstract

**Background:**

Voriconazole (VRCZ) has non-linear pharmacokinetics, so personalized dosing based on pharmacokinetics is important. Approximately 20% of Asians are poor metabolizers of CYP2C19. In Japan, challenges include rapid measurement of VRCZ blood levels, feedback of results to physicians, and rapid therapeutic drug monitoring (TDM). The aim of this study was to evaluate the rapid TDM implementation system for VRCZ in outpatients.

**Methods:**

Measurement of outpatient VRCZ TDM was started at Kochi medical school Hospital in January 2024 based on the blood concentration measurement flow by LM1010 high performance liquid chromatograph (HPLC-UV, Hitachi High-Tech Science Corporation). From January to April 2024, we compared the trough values of VRCZ and the reporting time of blood concentration measurement results after sample collection using the conventional externally requested measurement method (Method A) and the measurement method we constructed (Method B). In addition, we investigated the occurrence of hepatotoxicity from the perspective of preventing adverse events.

**Results:**

The trough concentration of VRCZ were 2.97±1.15 mg/L for method A (n=7) and 3.15±1.25 mg/L for method B (n=15) ,p=0.75. The time required to report VRCZ blood concentration measurement results was significantly shorter (Method A (n=7): 78.9±10.4 h vs. Method B (n=7): 0.44±0.02 h (p< 0.05). No hepatotoxicity was observed during treatment in TDM outpatients who underwent rapid VRCZ measurement.

**Conclusion:**

It is suggested that rapid determination of blood concentration of VRCZ by HPLC-UV will be an effective tool for TDM implementation and medical care support.

**Disclosures:**

**All Authors**: No reported disclosures

